# Tenecteplase Versus Alteplase in Acute Ischemic Stroke in Chinese Patients: Protocol for the ORIGINAL Study

**DOI:** 10.1161/SVIN.124.001363

**Published:** 2024-05-07

**Authors:** Xia Meng, Shuya Li, Hongguo Dai, Guozhi Lu, Weiwei Wang, Fengyuan Che, Yu Geng, Minghui Sun, Xiyan Li, Yongjun Wang

**Affiliations:** ^1^ Department of Neurology and Department of Clinical Trial Center Beijing Tiantan Hospital, Capital Medical University Beijing China; ^2^ China National Clinical Research Center for Neurological Diseases Beijing China; ^3^ Department of Emergency Linfen Central Hospital Linfen China; ^4^ Department of Neurology Hexigten Banner Mongolian Traditional Chinese Medicine Hospital Chifeng China; ^5^ Department of Neurology Xianyang Hospital of Yan'an University Xianyang China; ^6^ Department of Neurology Linyi People's Hospital Linyi China; ^7^ Center for Rehabilitation Medicine, Department of Neurology Zhejiang Provincial People's Hospital Hangzhou China; ^8^ Boehringer Ingelheim (China) Investment Co., Ltd Shanghai China

**Keywords:** alteplase, Chinese, functional outcome, ischemic stroke, symptomatic intracerebral hemorrhage, tenecteplase

## Abstract

**Background:**

Tenecteplase, a bioengineered variant of alteplase, is a new alternative thrombolytic agent. The ORIGINAL study aims to evaluate the efficacy and safety of tenecteplase versus standard care in Chinese patients with acute ischemic stroke within 4.5 hours of symptom onset.

**Methods:**

This is a multicenter phase III study that employs a randomized (1:1), active‐controlled, parallel‐group, prospective, open‐label, blinded–end point design. Adult participants (aged ≥18 years) with acute ischemic stroke who are eligible for intravenous thrombolysis within 4.5 hours of symptom onset according to current guideline recommendations are recruited from ≈55 neurology clinics/stroke centers throughout China.

**Results:**

The primary objective of the ORIGINAL study is to demonstrate the noninferiority of tenecteplase (0.25 mg/kg) to alteplase (0.9 mg/kg) on the basis of the proportion of patients who achieve a modified Rankin Scale score of 0 or 1 on day 90, that is, a favorable functional outcome. The prespecified noninferiority risk ratio margin is 0.937. Secondary end points include other functional outcomes and the following safety end points: adjudicated symptomatic intracerebral hemorrhage (up to 36 hours after the end of study drug administration) based on the European Cooperative Acute Stroke Study III definition; all‐cause death within 90 days; and the proportion of patients with a modified Rankin Scale score of 5 or 6 on day 90.

**Conclusions:**

It is anticipated that the results of this study will contribute to the growing body of evidence for the noninferiority of tenecteplase to alteplase given within 4.5 hours of acute ischemic stroke symptom onset and support a new indication for tenecteplase in China.

**Clinical Trial Registration Information:**

NCT04915729 (https://clinicaltrials.gov/study/NCT04915729)

**Nonstandard Abbreviations and Acronyms**

AE: adverse event
AIS: acute ischemic stroke
DMC: data monitoring committee
EAC: end point adjudication committee
ECASS: European Cooperative Acute Stroke Study
mRS: modified Rankin Scale
NIHSS: National Institutes of Health Stroke Scale
PROBE: prospective, open‐label, blinded–end point
sICH: symptomatic intracerebral hemorrhage

**Clinical Perspective**

- This article describes the design of the ORIGINAL study, which aims to demonstrate the noninferiority of tenecteplase (0.25 mg/kg; Boehringer Ingelheim) to alteplase, the current standard of care, in Chinese patients with acute ischemic stroke eligible for intravenous thrombolysis within 4.5 hours of symptom onset.
- Tenecteplase, a bioengineered variant of alteplase, is a new alternative thrombolytic agent that is administered as a single intravenous bolus without the need for an infusion.
- The ORIGINAL study is a multicenter phase III study that employs a randomized (1:1), active‐controlled, parallel‐group, prospective, open‐label, blinded–end point design.
- It is anticipated that the findings of this study will support a new indication for this drug in China and contribute to the body of evidence regarding the use of tenecteplase in patients with acute ischemic stroke.

The burden of stroke continues to increase globally.^1^ One in every 3 stroke‐related deaths worldwide occurs in China,^1^ where stroke is the leading cause of both death and disability combined.^2^ The majority (62%–87%) of incident strokes are ischemic in nature.^1^, ^3^ Alteplase, given as a bolus followed by a 1‐hour infusion, is the standard‐of‐care intravenous thrombolytic agent for treating eligible individuals with acute ischemic stroke (AIS) up to 4.5 hours after stroke onset.^4^, ^5^, ^6^

Tenecteplase is a tissue plasminogen activator produced by recombinant DNA technology, whereby the protein structure of alteplase is modified at 3 sites (T103N, N117Q, KHRR 296–299 AAAA), resulting in higher fibrin specificity and a longer half‐life, which are associated with practical advantages over alteplase.^7^, ^8^, ^9^ It has been indicated in the United States and in Europe as a first‐line thrombolytic agent for the management of eligible patients with acute ST‐elevated myocardial infarction when coronary intervention cannot be performed in a timely manner.^10^, ^11^, ^12^ There is a growing body of evidence from investigator‐initiated studies in Western populations comparing tenecteplase to alteplase in patients with AIS.^13^, ^14^, ^15^, ^16^, ^17^ These findings supported the inclusion of tenecteplase as a treatment option for eligible patients with AIS in international guidelines.^5^, ^6^ Recently, expedited recommendations for the use of tenecteplase in patients with AIS were released by the European Stroke Organisation, stating that tenecteplase 0.25 mg/kg is a safe and effective alternative to alteplase 0.9 mg/kg for patients with AIS of <4.5 hours’ duration.^6^ The European Stroke Association expert consensus also unanimously suggested that tenecteplase may be favored over alteplase because of its ease of administration as a single intravenous bolus without the need for an infusion, in addition to similar clinical benefits.^6^

We describe the design of the ORIGINAL study, a randomized, controlled, phase III study being undertaken to evaluate the efficacy and safety of tenecteplase (0.25 mg/kg; Boehringer Ingelheim) in Chinese patients with AIS within 4.5 hours of symptom onset who are eligible to receive thrombolysis per the standard of care for AIS.^18^, ^19^ At the time of study registration, evidence on the treatment effect of tenecteplase 0.25 mg/kg in Chinese patients with AIS was lacking. It is anticipated that data from this study will support a new label indication for this drug in China, expanding the treatment armamentarium for patients with AIS.

## Methods and Design

### Study Design

The data that support the findings of this study are available from the upon reasonable request. The ORIGINAL study is a phase III study that was undertaken to demonstrate the efficacy and safety of tenecteplase (Metalyse; Boehringer Ingelheim) relative to alteplase, both administered within 4.5 hours of the onset of AIS symptoms, in Chinese adults. The study uses a randomized, active‐controlled, parallel‐group, prospective, open‐label, blinded–end point (PROBE) design, and participants are recruited from ≈55 neurology clinics/stroke centers throughout China. All sites, which must have clinical research qualification per the International Conference on Harmonisation guidelines on good clinical practices, are selected on the basis of their ability to receive, identify, evaluate, treat, and/or refer patients with suspected stroke, as well as obtain access to stroke expertise when necessary for diagnostic or treatment purposes. These sites should be able to offer round‐the‐clock comprehensive stroke care to patients with AIS.

The study protocol is approved by the appropriate institutional review board/independent ethics committee at each participating study site and by the relevant competent authority. The study is conducted in compliance with the protocol and the ethical principles of the Declaration of Helsinki, and in accordance with the International Conference on Harmonisation guidelines on good clinical practices. All participants or their legally accepted representative are required to provide written, informed consent in accordance with good clinical practice and local legislation before enrollment in the study. An impartial witness can be called upon to attest the consent process if the participant cannot read. Procedures that are performed as part of routine clinical care before obtaining informed consent can be used for screening if they are carried out within the allotted time frame (after stroke onset and before drug administration). The Standard Protocol Items: Recommendations for Interventional Trials checklist that details the recommended items to be included in a clinical trial protocol is available in the Supplementary Material. This study is registered with ClinicalTrials.gov (NCT04915729).

### Study Population

Chinese adults (aged ≥18 years) are eligible for inclusion in the study if they have an AIS with a measurable neurological deficit, indicated by a National Institutes of Health Stroke Scale (NIHSS) score of 1 to 25 (inclusive), symptoms that are present for at least 30 minutes without significant improvement, and they are able to receive thrombolytic therapy within 4.5 hours of symptom onset. A noncontrast computed tomography is used at screening to exclude patients presenting with intracranial hemorrhage. To be eligible, individuals with an NIHSS score of <4 are required to have measurable deficit in motor power (motor function scores for the arms or legs ≥1). Individuals planned for endovascular thrombectomy are eligible to be enrolled in this study.

Individuals with any of the following attributes are excluded from the study:

- Evidence of intracranial hemorrhage on computed tomography, symptoms suggesting a subarachnoid hemorrhage (even in the presence of a normal computed tomography scan), or scans showing multilobar infarction (hypodensity greater than one‐third of cerebral hemisphere)
- Acute bleeding diathesis, including but not limited to:
  ○ A history of suspected intracranial hemorrhage, suspected subarachnoid hemorrhage from aneurysm, or central nervous system damage (ie, neoplasm, aneurysm, intracranial or spinal surgery)
  ○ A neoplasm associated with an increased risk of hemorrhage
  ○ A known genetic predisposition to bleeding or a significant bleeding disorder or a history of 1 in the previous 6 months
  ○ Received heparin in the previous 48 hours and with an activated partial thromboplastin time exceeding the upper limit of normal
  ○ Currently using vitamin K–based oral anticoagulants (warfarin) with a prolonged prothrombin time (>15 seconds, or international normalized ratio >1.7) or novel oral anticoagulants (apixaban, dabigatran, rivaroxaban) with an activated partial thromboplastin time or a prothrombin time above the upper limit of normal
  ○ Platelet count <100,000/mm^3^ at screening
  ○ Traumatic external heart massage, obstetrical delivery, or recent puncture of a noncompressive blood vessel (eg, subclavian or jugular vein puncture) within the previous 10 days
  ○ Documented ulcerative gastrointestinal disease during the previous 3 months, esophageal varices, arterial aneurysm, or arterial/venous malformations
  ○ Known disorder associated with a significantly increased risk of bleeding
- Bacterial endocarditis, pericarditis, or acute pancreatitis at screening
- Significant trauma or major surgery (based on the investigator's assessment) in the previous 3 months
- Severe uncontrolled arterial hypertension (systolic blood pressure >185 mmHg or diastolic blood pressure >110 mmHg)
- Blood glucose <50 mg/dL at screening (nonfasting, point‐of‐care glucose testing is acceptable)
- Must/expected to continue to take restricted medications or any drug considered likely to interfere with the safe conduct of the study
  ○ Restricted medications are any thrombolytic, other than the study drug, and oral anticoagulants, antiplatelet agents, or intravenous heparin within 24 hours following study drug administration
  ○ Low doses of subcutaneous heparin (<10 000 IU/24 hours or equivalent) can be given to prevent deep vein thrombosis in exceptional circumstances and if the activated partial thromboplastin time will not be prolonged to >2‐fold higher than baseline
  ○ Intravenous heparin can only be administered ≥24 hours after study drug administration following a second computed tomography scan to confirm the absence of intracranial hemorrhage

### Treatment, Randomization, and Blinding

A centralized, web‐based, real‐time interactive response technology system will be used to screen patients upon admission. Upon confirmation of eligibility, randomization is initiated through the system. The only laboratory result required before randomization is point‐of‐case testing of random glucose, which is immediate.

Patients are randomized (1:1) to receive either intravenous tenecteplase (0.25 mg/kg; maximum dose, 25 mg) administered as a bolus over 5 to 10 seconds, or intravenous alteplase (0.9 mg/kg; maximum dose 90 mg), with 10% of the dose administered as a bolus over 5 to 10 seconds and the remainder administered immediately as an infusion over 1 hour. Randomization is stratified by baseline NIHSS score (<6, 6–15, >15) and age (≤80 years, >80 years), and takes place in blocks using a pseudo‐random number generator.

According to the PROBE design, study drugs are given open label, while those responsible for the assessment of the primary efficacy end point are blinded to treatment allocation.

Administration of the study drugs occurs within 4.5 hours of symptom onset.

### Outcomes

The primary objective of the ORIGINAL study is to demonstrate the noninferiority of tenecteplase relative to alteplase, both administered within 4.5 hours of AIS symptom onset, in Chinese adults. The primary objective is analyzed on the basis of the proportions of patients who achieve a modified Rankin Scale (mRS) score of 0 or 1 on day 90, that is, a favorable functional outcome (primary end point).

Secondary efficacy end points are:

- Major neurological improvement at 24 hours (NIHSS score of 0 or at least a 4‐point improvement from baseline)
- mRS score of 0–2 on day 90
- Change in NIHSS score from baseline to day 90
- Distribution of mRS score on day 90
- Barthel Index of ≥95 on day 90

Secondary safety end points are:

- On‐treatment symptomatic intracerebral hemorrhage (sICH) based on the European Cooperative Acute Stroke Study (ECASS) III definition of sICH^20^
- Mortality within 90 days
- mRS score of 5 or 6 on day 90

Further functional end points, which were based on predefined cutoff points for the various functional measures, are listed in the Supplementary Material (Table S1), along with additional safety end points, including the frequency of adverse events (AEs). Both the investigator and adjudication committees evaluate and report on sICH events, and the events are blinded and adjudicated on an ongoing basis. sICH events are evaluated according to the ECASS III definition (secondary end point),^20^ as well as the Safe Implementation of Thrombolysis in Stroke–Monitoring Study and ECASS II definitions (further end points).^21^, ^22^, [Table svi212916-tbl-0001]

### Assessments

Baseline characteristics are collected, and clinical and laboratory assessments are undertaken at screening. Clinical assessments undertaken during the follow‐up are shown in Table. Follow‐up procedures include a noncontrast computed tomography scan at 22 to 36 hours upon initiating the study drug to identify any intracranial hemorrhage. Any incidence of intracranial hemorrhage identified by imaging will be referred to the end point adjudication committee (EAC) for adjudication of potential sICH, and the case is required to be reported as a serious AE. In the event of clinical deterioration, further optional computed tomography scans can be taken. NIHSS measurements are taken at 2 hours, 24 hours, 1 week, 1 month, and 3 months; mRS and Barthel Index at 1 month and 3 months; and the Glasgow Outcome Scale at 3 months. Assessments of mRS and NIHSS are performed by certified, trained neurologists blinded to treatment allocation. mRS assessors at follow‐up are masked to treatment allocation, as they are independent from those who enroll patients and administer treatments. AEs are recorded throughout the study and coded according to the Medical Dictionary for Regulatory Activities (version 26.0). AEs occurring up to 7 days after the end of study drug administration are considered on treatment. Owing to their very nature, certain AEs will be considered serious AEs irrespective of whether they meet the criteria for serious AEs, in accordance with the European Medicines Agency's initiative on important medical events. For example, certain hemorrhages, particularly those in internal organs, and hematomas with severe clinical consequences will always be considered serious AEs.

**Table svi212916-tbl-0001:** Assessments During the Follow‐Up Period (After Study Drug Administration)

Timing^*^	1±0.25 h	2±0.5 h	24±2 h	7±2 d/ discharge	30 (−2 or +7) d	90±7 d
Noncontrast CT			X^†^	(X)^‡^	(X)^‡^	
Physical examination			X			
Vital signs	X	X	X	X	X^‡^	X^‡^
Laboratory tests			X^‡^	X		X^‡^
12‐lead ECG				X		
NIHSS score^§^		X	X	X	X	X
Glasgow Outcome Scale						X
mRS score					X	X
Barthel Index					X	X
Adverse events	X	X	X	X	X	X

CT indicates computed tomography; ECG, electrocardiogram; mRS, modified Rankin Scale; and NIHSS, National Institutes of Health Stroke Scale.

* Relative to the start of study drug administration.

^†^ 22–36 h after the start of study drug administration.

^‡^ Optional (in cases of clinical deterioration or clinical demand [laboratory tests]; in cases where the visit is remote for vital signs).

^§^ Including distal motor function.

### Study Oversight

An independent and blinded EAC is established to adjudicate all sICH events on an ongoing basis. The EAC comprises specialists with proven relevant expertise and will meet regularly to discuss these events (every 15–18 events reported or as often as it deems necessary).

An independent data monitoring committee (DMC) is also established to monitor critical safety events and comprises physicians experienced in treating AIS and a statistician. The DMC reviews unblinded safety data (divided into 2 treatment groups, but treatment not disclosed) on the basis of the results of the EAC review. The DMC also monitors the progress of the study, focusing on safety outcomes, and carry out regular benefit–risk assessments, the results of which will guide their advice regarding the continuation of the study, with or without modifications, or the termination of the study.

The EAC and DMC are fully external and independent of the study investigators, sites, and sponsor.

### Statistical Analysis

A sample size of 1478 (≈739 individuals per treatment group) would provide 80% power to demonstrate noninferiority between tenecteplase and alteplase in terms of the proportion of patients who achieve an mRS of 0 or 1 on day 90, using a noninferiority margin of 0.937 and a type 1 error of 0.025 (1‐sided). This is based on an expected response rate of 63% with alteplase, as published,^23^, ^24^ and 65.8% for tenecteplase, a conservative estimate based on an unadjusted absolute relative risk of 1.06 for tenecteplase versus alteplase in the AcT (Alteplase compared to Tenecteplase) study,^17^ which equates to a response rate of 66.8% for tenecteplase.

The noninferiority margin of 0.937 is based on the results of a meta‐analysis of data from randomized trials of intravenous alteplase to treat AIS,^25^ in which the unadjusted risk ratio (RR) for achieving an mRS score of 0 or 1 versus placebo was 1.24 (95% CI, 1.14–1.36). Per guidance from the United States Food and Drug Administration,^26^ taking the lower bound of the 95% CI (1.14) as M1 and f = 0.5 (to preserve at least 50% of the effect of alteplase), M2 is 1.0677. Therefore, the noninferiority margin (1/M2) for the RR is 0.937.

The comparative proportions of patients who achieve an mRS score of 0 or 1 on day 90 in each treatment group (primary end point) are analyzed according to the intention‐to‐treat principle using data from the full analysis set, that is, all individuals randomized and who received any dose of the study drug; data are analyzed according to the randomized treatment groups. A log‐binomial regression model adjusted for continuous covariates (baseline NIHSS score, age, and time to drug administration) is applied and transformed into RRs with respective 95% CIs. The multiple imputation approach is used for missing data. Noninferiority is indicated by a lower bound of the 95% CI for the RR for tenecteplase versus alteplase of >0.937. If the null hypothesis for noninferiority of tenecteplase versus alteplase is rejected, superiority testing will be conducted on the basis of the 2‐sided 95% CI for the RR in those who achieve an mRS score of 0 or 1 on day 90. Superiority is indicated by a lower bound of the 95% CI for the RR for tenecteplase versus alteplase of >1. Sensitivity analyses of the primary end point are undertaken using the last observation carried forward method to impute missing data for surviving patients in the full analysis set. Missing data at day 90 are replaced by the last observation carried forward for patients with posttreatment mRS data available, and by an mRS score of 5 (worst case excluding death) for those without. Additional sensitivity analyses are performed using observed cases in the full analysis set, and with the log‐binomial regression model including treatment only (unadjusted RR and 95% CI) and the continuous covariates (adjusted RR and 95% CI).

The primary end point is analyzed in the full analysis set in predefined subgroups on the basis of baseline NIHSS score (<6, 6–15, >15 and <4, ≥4), baseline age (≤80 years, >80 years), time to study drug administration from onset of stroke symptoms (≤3 hours, >3 hours), sex (male, female), atrial fibrillation (yes, no), diabetes (yes, no), and whether a thrombectomy is performed (yes, no). The log‐binomial model is fitted to each subgroup separately by modeling the primary end point with treatment, subgroup, and treatment‐by‐subgroup interaction terms. The multiple imputation approach is used to handle missing data.

Supplementary analyses of the primary end point are undertaken using data from the per protocol set (patients included in the full analysis set who do not have any important protocol deviations that may affect the evaluation of the primary end point). The analyses use the same statistical models in the primary analysis, employing the multiple imputation method to impute missing data and the observed cases approach previously described. Additional supplementary analyses are described in the Supplementary Material.

Analyses of secondary and further efficacy end points are exploratory in nature. Responder analyses (secondary efficacy end points of major neurological improvement at 24 hours [NIHSS score of 0 or at least a 4‐point improvement from baseline], an mRS score of 0–2 on day 90, and a Barthel Index of ≥95 on day 90) are undertaken with the same log‐binomial regression model used for the primary analysis of the primary end point in the full analysis set. Categorical treatment and continuous covariates are fitted to the binary response to calculate the RR with 95% CI for tenecteplase versus alteplase.

The secondary efficacy end point, change in NIHSS score from baseline to day 90, is analyzed using a restricted maximum likelihood–based mixed model for repeated measures approach in the full analysis set, assuming that data are missing at randomization. The distribution of mRS on day 90 (secondary efficacy end point) is summarized descriptively, and a comparison of ordinal mRS on day 90 between the 2 treatment groups is carried out using an ordinal logistic regression model including the continuous covariates. The secondary safety end point, the proportion of patients with on‐treatment sICH in each treatment group according to the ECASS III definition, is compared between the groups using the Suissa–Shuster test with no imputation for missing data in the safety set (ie, all patients who were randomized and received the study drug; data will be analyzed according to the treatment received). The Chan and Zhang method is used to calculate the 95% CI for the unadjusted RR. Mortality at day 90 is compared between the treatment groups through a χ^2^ test with no imputation for missing data in the safety set, and the Wald method is used to provide the CI for the unadjusted RR. The proportions of patients with an mRS score of 5 or 6 on day 90 are analyzed with the log‐binomial regression model adjusted for continuous covariates in the full analysis set. Analysis of further safety end points are described in the Supplementary Material.

If the log‐binomial regression model fails to converge and provide a valid RR for any analysis in which it is used, a modified Poisson regression model will be used. For analyses of functional outcomes, data missing because of death are replaced by the worst scores, that is, 6 for the mRS, 42 for the NIHSS, 0 for the Barthel Index, and 5 for the Glasgow Outcome Scale.

## Discussion

The ORIGINAL study is being undertaken to provide robust evidence from a randomized, controlled, phase III study to support the use of tenecteplase in Chinese patients with AIS presenting within 4.5 hours of symptom onset; such data were not available at the time of study registration.

The ORIGINAL study is a classical randomized trial, with centralized adjudication of sICH events by an EAC and the regular review of safety data by the DMC. The ORIGINAL study comprises an inclusive population of patients to reflect those in clinical practice who are eligible for intravenous thrombolysis. This is consistent with the label for alteplase, which includes recommendations for thrombolysis in patients with mild AIS with functional disability and in those for whom endovascular thrombectomy is planned.^19^ Since recent evidence have also demonstrated the positive benefit–risk profile of alteplase versus placebo in patients with AIS aged ≥80 years,^27^ this population was also included in this study. Overall, the AIS population included in this trial is aligned with that recommended for thrombolysis by international guidelines.^5^, ^6^, ^18^ It is anticipated that the results of this classical phase III, randomized, controlled study will add to the existing body of evidence supporting the use of tenecteplase in patients with AIS. This evidence includes the results of the phase III, registry‐linked, randomized, controlled AcT study conducted in Canada, in which a pragmatic approach was taken by which data collection was linked to registries and a simplified randomization procedure was used in addition to deferred informed consent.^17^, ^28^ This evidence also includes the results of another recent phase III, PROBE study, TRACE‐2 (Tenecteplase Versus Alteplase in Acute Ischaemic Cerebrovascular Events; tenecteplase: Recomgen), which was since conducted in China, in which patients eligible for endovascular thrombectomy and those who had a minor stroke were excluded.^29^

The ORIGINAL study uses a PROBE design. While a double‐blind, double‐dummy design is ideal for phase III, randomized, controlled studies, this would significantly increase the complexity of study drug administration and may have ethical implications. While study drugs are given open label, according to the PROBE design, those involved in assessing the primary end point (mRS score of 0 or 1 at 90 days) remain blinded to study drug allocation.

In conclusion, the ORIGINAL study was undertaken to demonstrate the noninferiority of tenecteplase to alteplase in Chinese patients with AIS who are eligible for intravenous thrombolysis within 4.5 hours of symptom onset. It is anticipated that the findings of this study will support a new indication for this drug in China and contribute to the body of evidence regarding the use of tenecteplase in patients with AIS.

## Author Contributions

X. Meng, S. Li, H. Dai, G. Lu, W. Wang, F. Che, Y. Geng, and Y. Wang are study investigators. The authors were responsible for all content and editorial decisions, were involved at all stages of development, and approved the final version of the manuscript. The authors met the criteria for authorship as recommended by the International Committee of Medical Journal Editors. The authors did not receive payment related to the development of the manuscript. Boehringer Ingelheim (China) Investment Co., Ltd was given the opportunity to review the manuscript for medical and scientific accuracy, as well as intellectual property considerations.

## Sources of Funding

This work is supported and funded by Boehringer Ingelheim (China) Investment Co., Ltd. Boehringer Ingelheim Pharma GmbH & Co. KG manufactures and provides the study drug (tenecteplase).

## Disclosures

Minghui Sun and Xiyan Liu are employees of Boehringer Ingelheim (China) Investment Co., Ltd. All other authors have no competing interests to declare.

## Supporting information



Table S1. Further endpoints in the ORIGINAL study
S1. Additional statistical analyses
