# Supplementary material for: Tenecteplase Versus Alteplase in Acute Ischemic Stroke in Chinese Patients: Protocol for the ORIGINAL Study
Source: Stroke Vasc Interv Neurol. 2024 May 7;4(4):e001363. doi: 10.1161/SVIN.124.001363 (PMC12778536; doi:10.1161/SVIN.124.001363)
Supplement: Supplementary file 1 — Table S1. Further endpoints in the ORIGINAL study S1. Additional statistical analyses [file SVI2-4-e001363-s001.pdf]

SUPPLEMENTAL MATERIALS

Table S1. Further endpoints in the ORIGINAL study

**Efficacy**
• Barthel Index score of ≥85 on day 90<br>• Glasgow Outcome Scale score of 1 on day 90<br>• 8-point improvement in NIHSS score from baseline or NIHSS score of 0 or 1 on day 30<br>• mRS score of 0 or 1 on day 30<br>• mRS score 0–2 on day 30<br>• Barthel Index score of ≥95 on day 30<br>• NIHSS score at 2 and 24 hours and on days 8 and 30<br>• 4-point improvement in NIHSS score from baseline or NIHSS score of 0 or 1 on day 8
**Safety**
• On-treatment sICH (up to 36 hours after the end of study-drug administration), based on the SITS-MOST definition of sICH[1]<br>• On-treatment sICH (up to 7 days after the end of study-drug administration), based on the ECASS II definition of sICH[2]<br>• Frequency and severity of adverse events<br>• Time to death (cut-off 90 days)

ECASS II indicates European Cooperative Acute Stroke Study II; mRS, modified Rankin Scale; NIHSS, National Institutes of Health Stroke Scale; sICH, symptomatic intracranial hemorrhage; SITS-MOST, Safe Implementation of Thrombolysis in Stroke – Monitoring Study

S1. Additional statistical analyses

A supplementary analysis of the primary endpoint will be undertaken, utilizing a logistic regression model adjusted for the continuous covariates (baseline National Institutes of Health Stroke Scale [NIHSS] score, age, and time to drug administration) to estimate the marginal risk difference between the treatment groups using a marginal standardization method. This analysis will be undertaken in the full analysis set (FAS; all individuals randomized and who received any dose of study drug – data will be analysed according to the randomized treatment groups). SAS® macro '%Margins' will be used to estimate marginal mean and population average treatment effects. The delta method will be used to calculate the confidence intervals.

S1.1 Analysis of further endpoints

All further endpoints will be considered exploratory in nature. The efficacy endpoints will be analysed using a responder analysis in the FAS:

- Barthel Index score of ≥95 on day 30
- Barthel Index score of ≥85 on day 90
- Glasgow Outcome Scale score of 1 on day 90
- Eight-point improvement in NIHSS score from baseline or NIHSS score of 0–1 on day 30
- modified Rankin Scale (mRS) score of 0 or 1 on day 30
- mRS score of ≤2 on day 30
- 4-point improvement in NIHSS score from baseline or NHSS score of 0–1 on day 8

The same log-binomial regression model used for primary analysis of the primary endpoint, adjusted for baseline NIHSS score, age, and time to drug administration since onset of stroke symptoms, will be fitted to the corresponding binary response variable to calculate the estimated risk ratio (RR) for the tenecteplase group versus the alteplase group. For the mRS scores on day 30, missing data will be imputed using the multiple imputation method. For all other endpoints, analysis will be based on observed cases. If the log-binomial regression model fails to converge and provide a valid RR, a modified Poisson regression model will be used.

The further safety endpoints will be analyzed in the safety set (ie all patients who were randomized and received the study drug – data will be analyzed according to treatment received). On-treatment sICH based on the SITS-MOST and ECASS II definitions will be analyzed in the same manner as the secondary sICH safety analysis.

Time to death will be analyzed with Kaplan–Meier survival probability estimates and compared using a log-rank test with no imputation of missing data. Data will be censored from day 90.
